# Methylene blue reduces monoamine oxidase expression and oxidative stress in human cardiovascular adipose tissue

**DOI:** 10.1007/s11010-024-05092-z

**Published:** 2024-08-21

**Authors:** Oana-Maria Aburel, Laurențiu Brăescu, Darius G. Buriman, Adrian P. Merce, Anca M. Bînă, Claudia Borza, Cristian Mornoș, Adrian Sturza, Danina M. Muntean

**Affiliations:** 1 Chair of Pathophysiology, Department III, “Victor Babeş” University of Medicine and Pharmacy of Timișoara, E. Murgu Sq. No. 2, 300041 Timişoara, Romania; 2 Centre for Translational Research and Systems Medicine, “Victor Babeş” University of Medicine and Pharmacy of Timișoara, E. Murgu Sq. No. 2, 300041 Timişoara, Romania; 3 Doctoral School Medicine-Pharmacy, “Victor Babeş” University of Medicine and Pharmacy of Timișoara, E. Murgu Sq. No. 2, 300041 Timişoara, Romania; 4 Department VI, Clinic of Cardiovascular Surgery, “Victor Babeş” University of Medicine and Pharmacy of Timișoara, E. Murgu Sq. No. 2, 300041 Timişoara, Romania; 5 Institute for Cardiovascular Diseases, G. Adam Str. No.13A, 300310 Timișoara, Romania; 6 Department VI, 2nd Clinic of Cardiology, “Victor Babeş” University of Medicine and Pharmacy of Timișoara, E. Murgu Sq. No. 2, 300041 Timişoara, Romania; 7 Timișoara County Hospital, 156 L. Rebreanu Str, 300723 Timişoara, Romania

**Keywords:** Epicardial and perivascular adipose tissue, Heart failure, Monoamine oxidases, Oxidative stress, Methylene blue

## Abstract

Cardiovascular diseases represent the major cause of morbidity mainly due to chronic heart failure. Epicardial (EAT) and perivascular adipose tissues (PVAT) are considered major contributors to the pathogenesis of cardiometabolic pathologies. Monoamine oxidases (MAOs) are mitochondrial enzymes recognized as sources of reactive oxygen species (ROS) in cardiometabolic pathologies. Methylene blue (MB) is one of the oldest protective agents, yet no data are available about its effects on adipose tissue. The present pilot study was aimed at assessing the effects of MB: (i) on MAO expression and (ii) oxidative stress in EAT and PVAT harvested from patients with heart failure subjected to cardiac surgery (*n* = 25). Adipose tissue samples were incubated with MB (0.1 µM/24 h) and used for the assessment of MAO gene and protein expression (qPCS and immune fluorescence) and ROS production (confocal microscopy and spectrophotometry). The human cardiovascular adipose tissues contain both MAO isoforms, predominantly MAO-A. Incubation with MB reduced MAOs expression and oxidative stress; co-incubation with serotonin, the MAO-A substrate, further augmented ROS generation, an effect partially reversed by MB. In conclusion, MAO-A is the major isoform expressed in EAT and PVAT and contribute to local oxidative stress; both effects can be mitigated by methylene blue.

## Introduction

Coronary heart disease (CHD) is the leading cause of mortality due to myocardial infarction and morbidity due to heart failure [1]; with the aging of the population and increased number of hospitalizations, the latter is currently a major challenge for the healthcare systems worldwide [2].

Epicardial adipose tissue (EAT), the fat depot on the myocardial surface being in direct contact with the coronary arteries, has been widely recognized for more than one decade as a biologically active organ whose volume has emerged as quantifiable risk factor for CHD, particularly in the setting of obesity [3]. Indeed, the expansion of EAT under obesogenic conditions is promoting the development of atherosclerosis (primary in coronary arteries surrounded by it) via the activation of local inflammation and apoptosis and also acting as metabolic transducer within the paracrine crosstalk with the subjacent myocardium (excellently reviewed in refs. [4, 5]). More recently, EAT dysfunction has emerged as a central pathomechanism and therapeutic target for both heart failure with preserved ejection fraction (HFpEF) and atrial fibrillation in patients with cardiometabolic diseases and chronic systemic inflammation [6].

Perivascular adipose tissue (PVAT), the fat depot surrounding arteries with crucial roles in regulation and maintenance of vascular tone and endothelial function, expresses an inflammatory phenotype in obesity and is responsible, together with the EAT, for the emergence of the so-called “outside-to-inside” model of inflammation in CHD [7, 8].

Increased oxidative stress is another pathomechanism responsible for the dysfunction of the epicardial and perivascular adipose pools. One potential source of reactive oxygen species (ROS) is monoamine oxidases (MAO) with two isoforms, MAO-A and MAO-B at the outer mitochondrial membrane. We have firstly reported the presence of both MAOs in the PVAT surrounding the mammary arteries isolated from patients with CHD undergoing revascularization procedures [9]. Whether the enzyme is also expressed in the EAT and PVAT surrounding the large arteries it is not known.

Methylene blue (MB, 3,7-bis (dimethylamino)-phenazathionium chloride), a more than 100 years-old synthetic compound, was approved by FDA for the treatment of methemoglobinemia, cyanide poisoning and malaria and has recaptured the researchers attention for its neuroprotective properties in neurodegenerative disorders, ischemic and traumatic brain injury via anti-apoptotic, anti-inflammatory, and anti-oxidant effects besides the increased energetic metabolism, since it acts as an alternative electron carrier in the case of the dysfunctional electron transport system (ETS). Also, it acts as a potent antidepressant due to its preferential accumulation in the brain where it readily penetrates neuronal mitochondria, inhibits monoamine oxidase (MAO) and activates signaling pathways involved in mitochondrial biogenesis and autophagy [10, 11]. More recently, MB has been reported to protect the rat kidney mitochondria from cisplatin-induced cytotoxicity by increasing the expression of genes involved in the mtDNA repair pathway [12]. We have previously demonstrated that MB improved mitochondrial respiration in rat heart mitochondria isolated from diabetic and non-diabetic animals [13] and alleviated endothelial dysfunction together with a reduction in oxidative stress in aortas harvested from diabetic rats [14].

We hypothesized that MAOs are expressed in human EAT and PVAT, contribute to the oxidative stress, and can be modulated by MB.

## Materials and methods

This study conforms with the ethical principles for medical research involving human subjects outlined in the Declaration of Helsinki. The Commission for Research Ethics of “Victor Babeș” University for Medicine and Pharmacy of Timişoara and the Commision for Ethics in Research and Development of the Institute for Cardiovascular Diseases of Timișoara (no. 371/20.01.2021) approved the study protocol. Written informed consent was obtained from all patients prior to surgery.

Adipose samples were harvested from 25 patients with indication for elective heart surgery for cardiac pathologies (valvular disorders and coronary heart disease) and heart failure (HF) with either mildly reduced or preserved left ventricle ejection fraction (*LVEF* = 47.7% ± 6.23). Patients’ characteristics and preoperative medication were collected from medical records and are presented in Table 1.

**Table 1:** Characteristics of the study group

Demographics Age Sex, M/F (male/female)	64.5 ± 7.9 20/5
Clinical characteristics BMI Cholesterol (mg/dL) FPG LVEF Valvulopathies CHD HT AF AFL	27.5 ± 5 177 ± 46.8 104.4 ± 19 47.7 ± 6.23 15 (60) 10 (40) 15 (60) 2 (8) 1 (4)
Preoperative medication Aspirin β-Blockers Anticoagulants Statins Nitrates Calcium channel blockers Diuretics Insulin Oral antidiabetics Antibiotherapy	14 (56) 18 (72) 3 (12) 17 (68) 7 (28) 8 (32) 25 (100) 3 (12) 2 (8) 0

Data are presented as means ± S.E.M. In parentheses are the percentages of the corresponding variable. *BMI* body mass index, *FPG* fasting plasma glucose, *LVEF* left ventricular ejection fraction, *CHD* coronary heart disease, *HT* hypertension, *AF* atrial fibrillation, *AFL* atrial flutter

### Preparation of samples

EAT samples were harvested from the anterior wall of the right ventricle, while PVAT samples from peri-aortic and peri-pulmonary artery adipose tissue. EAT and PVAT samples were placed in an ice-cold buffer containing: 10 mM Ca-EGTA (ethylene glycol tetraacetic acid), 0.1 μM free calcium, 20 mM imidazole, 20 mM taurine, 50 mM K-MES (2-(N-morpholino)ethanesulfonic acid), 0.5 mM DTT (dithiothreitol), 6.56 mM MgCl_2_, 5.77 mM ATP (adenosine-5'-triphosphate), 15 mM phosphocreatine and immediately transferred to the laboratory, as previously described [15]. Adipose tissue samples were incubated for 24 h at 37 °C with or without 0.1 µM MB, then snap-frozen for further experimental procedures.

### Assessment of MAO A and B expression

Both gene and protein expressions of MAO isoforms were determined according to previously described techniques [16]. In order to assess the gene expression of both MAO isoforms in adipose tissues total RNA was isolated (with the Aurum Total RNA Mini Kit, Biorad) and used for reverse transcription (with the iScript Advanced cDNA Synthesis Kit, Biorad). MAO-gene expression was evaluated by quantitative real time polymerase chain reaction (qRT-PCR), in the presence vs. the absence of MB (0.1 µM, 24 h incubation period). The sequence information used from the NCBI database to design the primers against MAOs was as follows: (5'- > 3')—human MAO-A fw AGG ACT ATC TGC TGC CAA AC; human MAO-A rev AAG CTC CAC CAA CAT CTA CG; human MAO-B fw GAA GAG TGG GAC AAC ATG AC; human MAO-B rev CTC CAC ACT GCT TCA CAT AC). The housekeeping gene and its primers were as follows: GADPH (fw): 5' CTC ATG ACC ACA GTC CAT GC -3'and GADPH (rv): 5'- TTC AGC TCT GGG ATG ACC TT -3', respectively.

Protein expression of MAOs isoforms was quantified in frozen sections of EAT and PVAT using both MAO-A (Abcam, ab126751) and MAO-B (Abcam, ab175136) primary antibodies and a secondary goat anti-rabbit antibody Alexa Fluor labeled (Invitrogen, A32731). Nuclei were stained with DAPI (Santa Cruz, SC3598). The slides were analyzed in confocal microscopy (Olympus Fluoview FV1000 confocal microscope) by means of the Image J software.

### Evaluation of oxidative stress

ROS production in the adipose tissue samples was determined in the presence vs. the absence of MB (0.1 µM, 24 h incubation period) using 2 previously described techniques, immune fluorescence (IF) [17] and spectrophotometry [18], respectively.

Assessment of oxidative stress by means of IF used the dihydroethidium probe (DHE, Sigma-Aldrich-Merck). The superoxide indicator DHE, exhibits blue-fluorescence in the cytosol until oxidized, where it intercalates within the cells’ DNA, staining the nuclei a bright fluorescent red. Briefly, the adipose tissue fragments embedded and frozen in the optimal cutting temperature compound** (**OCT) were cut in 8 µm cryosections (Slee, MTC, Mainz) and placed on glass slides. After 3 washes with PBS, cryosections were incubated with DHE at room temperature. The slides were mounted with Vectashield (Vector Laboratories) and assessed in confocal microscope (Olympus Fluoview FV1000). Images were obtained using laser excitation at 488 nm 488 nm and emission at 610 nm and analyzed with the above mentioned software (Image J).

Hydrogen peroxide production was assessed in EAT and VAT samples by means of the Ferrous iron xylenol orange OXidation (FOX) assay (PeroxiDetect Kit, Sigma Aldrich Merck). The principle of the assay is that in the presence of peroxides, ferrous iron (Fe^2+^) is oxidized to the ferric (Fe^3+^) iron; the latter ion will then form a colored adduct with xylenol orange (3,3′-bis-N,N-bis(carboxymethyl)aminomethyl-o-cresolsulfonephthalein, sodium salt) that is measured at 560 nm. The H_2_O_2_ production is then calculated using a standard curve and results are expressed in nmol H_2_O_2_/mg tissue/h.

## Chemicals

All reagents were purchased from Sigma Aldrich Merck unless otherwise stated.

## Data analysis

Data analysis was performed by GraphPad Prism version 9.0 for Windows (GraphPad Software, USA). Data were expressed as mean ± S.E.M. Student t test, and for multiple comparisons, one-way ANOVA followed by Bonferroni post hoc analysis were used, differences between groups being considered significant at *p* < 0.05.

## Results

### MAO-A is the major isoform in human eat and pvat and both isoforms expression was reduced by acute incubation with methylene blue

**Fig. 1:**
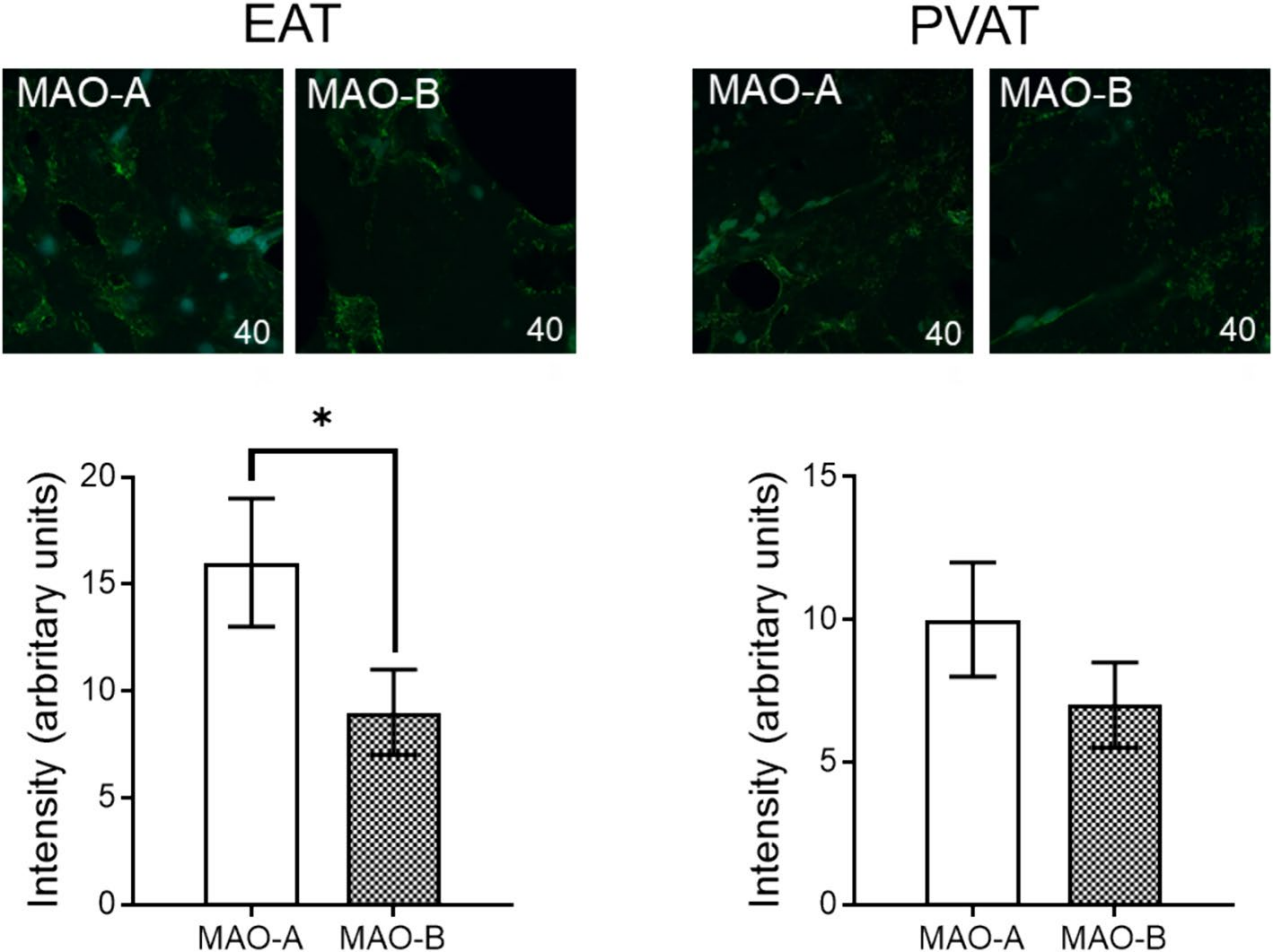
MAO protein expression in human EAT and PVAT samples. (*IF* intensity, *n* = 25, values are means ± S.E.M; *p < 0.05)

Our data showed that both MAO isoforms are present in human EAT and PVAT, with a higher expression in the former as compared to the latter (by analyzing the IF intensity). However, the protein expression of MAO-A isoform was significantly higher.

as compared to the one of MAO-B (Fig. 1).

**Fig. 2:**
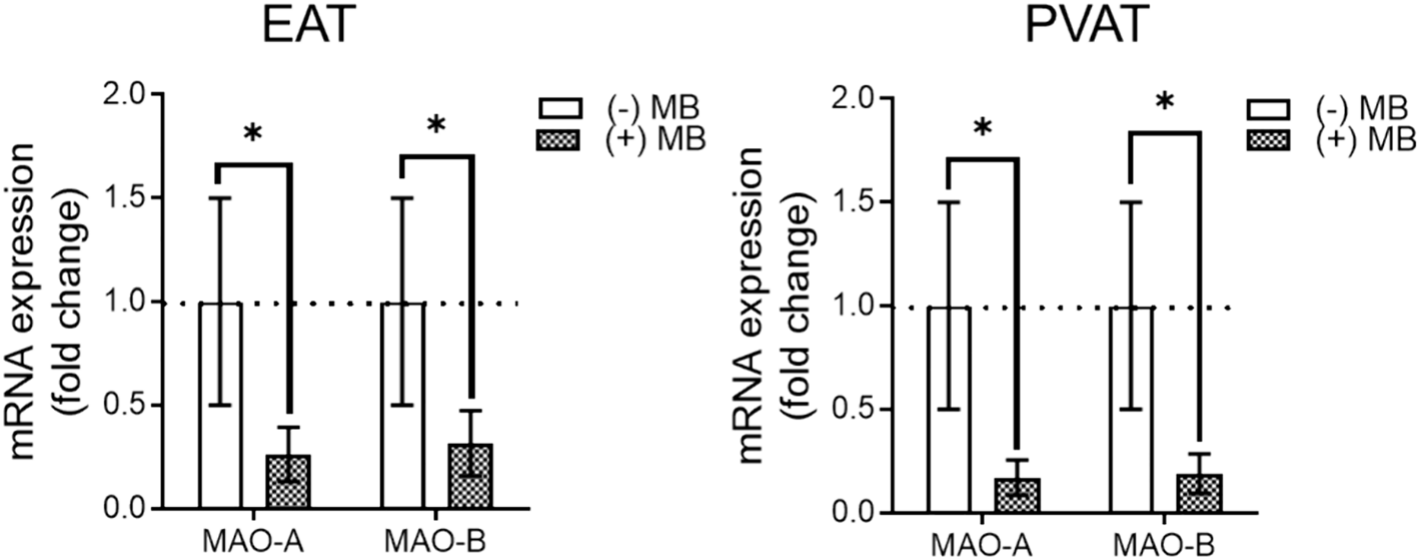
MAOs gene expression in human EAT and PVAT samples in the presence *vs.* the absence of MB. (mRNA—fold increase for MAO-A and MAO-B relative to the housekeeping gene GADPH (*n* = 25; values are means ± S.E.M; *p < 0.05)

The gene expression of MAOs in EAT and PVAT was further assessed by RT-PCR, in the presence vs. the absence of MB. Acute incubation of the samples with MB (0.1 µM, 24 h) significantly decreased the expression of both MAO isoforms in human adipose tissue (Fig. 2).

## Acute incubation with mb mitigated oxidative stress in human EAT and PVAT

**Fig. 3:**
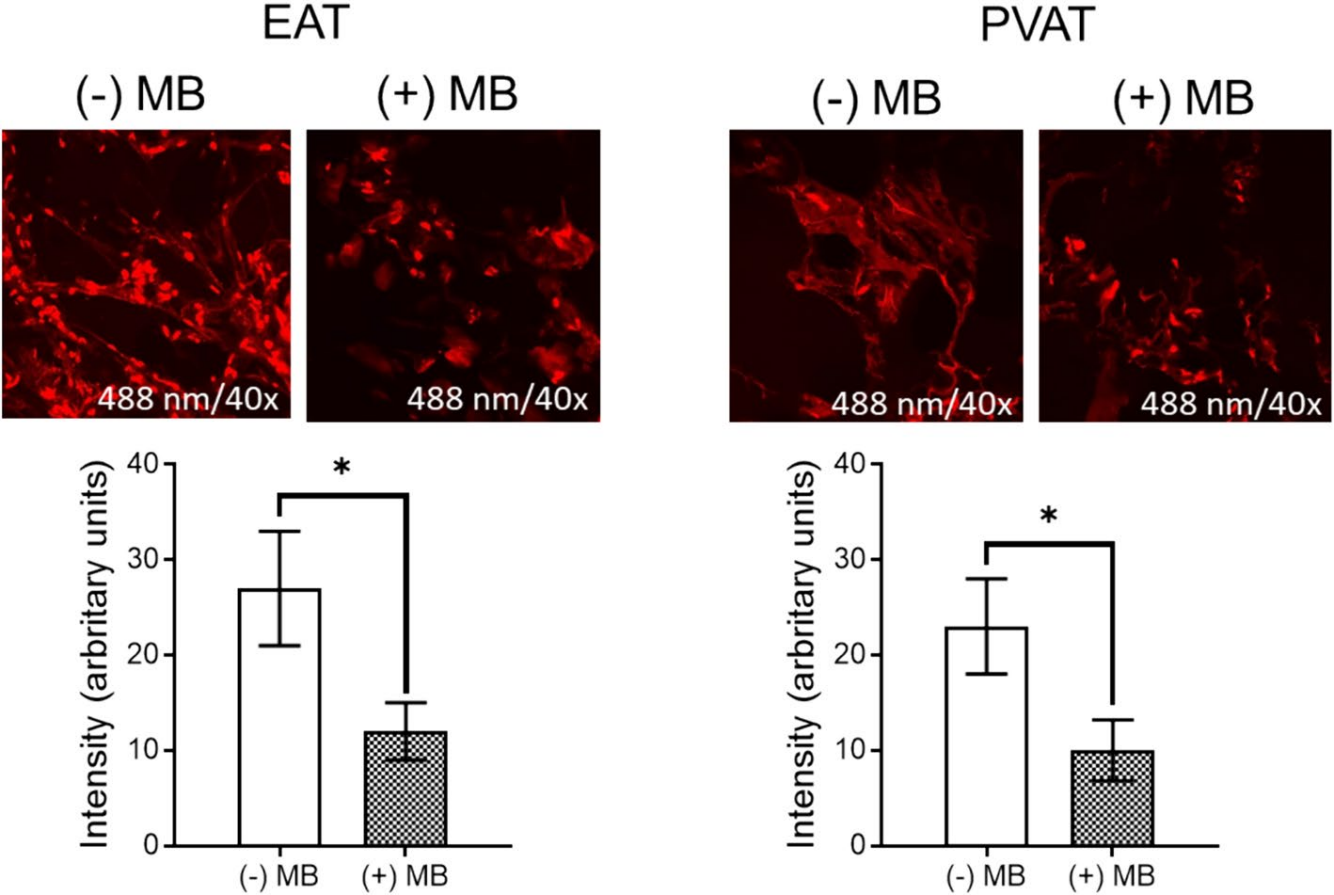
ROS assessment with DHE stain in human EAT and PVAT in the presence *vs* absence of MB. (fluorescence intensity, *n* = 25, values are means ± S.E.M; *p < 0.05)

Since the increased oxidative stress is a classic feature of all dysfunctional adipose tissue, ROS production in EAT and PVAT was assessed using the DHE probe and measured as a fluorescent red staining in confocal microscopy (Fig. 3).

As showed in Fig. 3, a higher intensity of the DHE stain (arbitrary units) was present in EAT as compared to PVAT, a result that recapitulates the observation on MAOs expression in the two types of adipose tissues presented in Fig. 1.

Ex vivo incubation with methylene blue (0.1 µM, 24 h) elicited a significant and comparable ROS reduction in both EAT and PVAT, confirming its antioxidant property in human cardiovascular adipose tissue (Fig. 3).

**Fig. 4:**
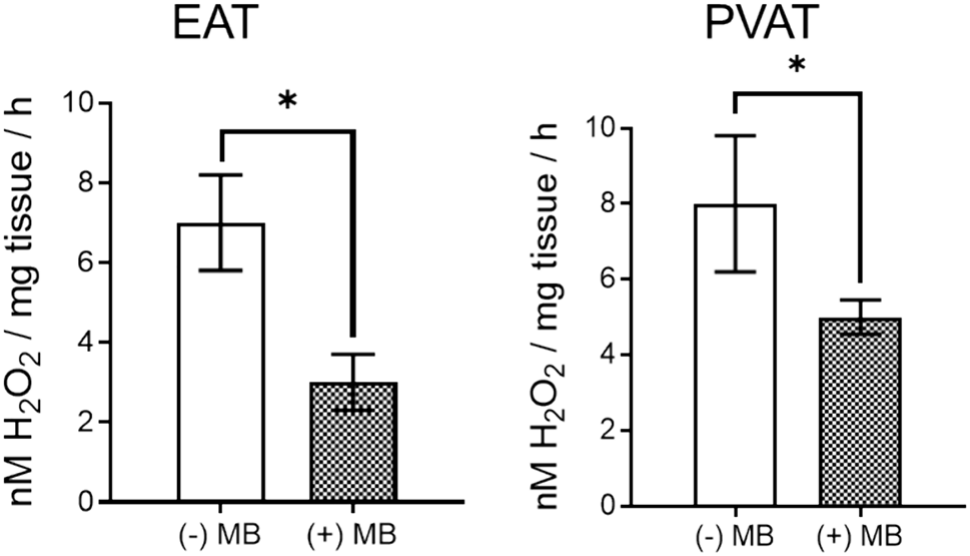
ROS assessment with FOX assay in human EAT and PVAT in the presence *vs* absence of MB. (H_2_O_2_ production in nM/mg/h, *n* = 25; values are means ± S.E.M; *p < 0.05)

Since DHE is classically regarded as a superoxide probe, we further determined the H_2_O_2_ production in EAT and PVAT by means of FOX assay, in the presence vs. the absence of methylene blue. As shown in Fig. 4, H2O2 production was significantly reduced in the presence of MB in both types of adipose tissues, apparently in a higher degree in EAT as compared to PVAT. Since MAO is a constant source of H_2_O_2_ and its expression was mitigated by MB particularly in EAT observation we might speculate on the role of MAOs as important source of oxidative stress in diseased human adipose tissue.

**Fig. 5:**
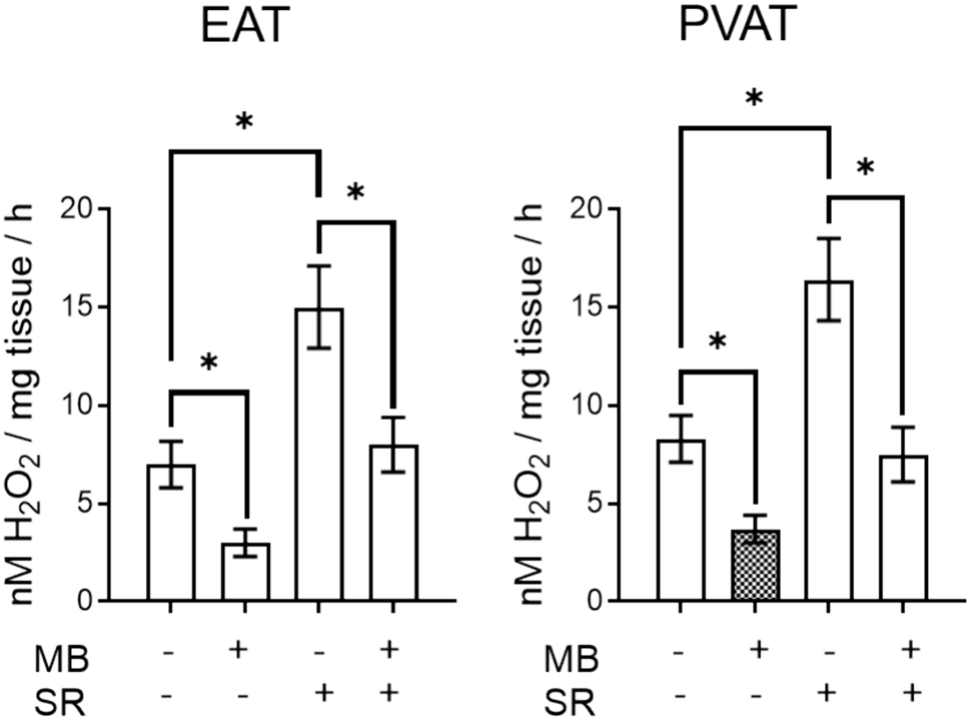
ROS assessment with FOX assay in human EAT and PVAT incubated or not with serotonin (SR) in the presence *vs* absence of MB. (H_2_O_2_ production in nM/mg/h, *n* = 25; values are means ± S.E.M; *p < 0.05)

Finally, in order to confirm that MAO-A, the major isoform in human adipose tissue, can be targeted with MB, we recapitulated the experiments in the presence vs the absence of serotonin (10 µM), the MAO-A substrate. Incubation with SR more than doubled the H_2_O_2_ production in both types of adipose tissue, an effect that was partially reversed by MB (Fig. 5). Since the H_2_O_2_ values in the presence of SR and MB did not reach the ones obtained with MB alone, we might speculate that other ROS sources contribute to the hydrogen peroxide generation and might be induced by SR (or even MB might be responsible for the generation of H_2_O_2_ in small amounts).

## Discussion

In the present study we recruited cardiac patients with HFpEF and HFmrEF referred for open-heart surgery, since it has been reported in the literature that EAT accumulation was associated with adverse prognosis in these categories of patients [19].

Over the past decade, both epicardial and perivascular adipose pools have been increasingly acknowledged as modulators of progression of cardiometabolic diseases (associated with chronic inflammation and insulin resistance) and pharmacological therapeutic targets [7, 20]. Both tissues are major sources of pro-inflammatory cytokines and growth factors that exert deleterious effects on both heart and vessels by paracrine signaling via direct diffusion. In particular, EAT isolated from obese, but also from overweight patients was found to have higher expression and levels of pro-inflammatory cytokines (IL-1, IL-6, TNF-α, and IFN-γ) than the paired subcutaneous fat [21]. Of note, in the studied group, BMI was 27.7 ± 4.9 (see Table 1) and only 3 patients presented a BMI over 30 (data not shown). We acknowledge as a limitation of our study the lack of measurement of serum inflammatory cytokines and markers (e.g., CRP) and of cardiac injury markers reported to be increased in the settings of cardiopulmonary bypass [22]*.* Also, this pilot study included more males (20) as compared to females (5); whether sex differences occur in response to MB is worth further investigation.

Moreover, EAT accumulation and its inflammation promote an arrhythmogenic substrate via fibrotic remodeling [23]. Left atrial myocardial fibrosis was positively correlated with the level of proinflammatory and profibrotic cytokines/chemokines, IL‐6, MCP‐1, and TNF‐α, in EAT [24] and myocardial fibrosis is a known substrate for atrial fibrillation. In the present study, 2 patients had atrial fibrillation and one atrial flutter (see Table 1) albeit a causal relation cannot be affirmed.

This pilot study was purported to assess both MAO expression as novel source of oxidative stress in EAT and PVAT samples, as well as the possibility to counteract it by acute exposure to MB. The first major finding is that MAO-A is the predominant isoform expressed in human cardiovascular adipose tissue that can be targeted (at least ex vivo) with submicromolar concentrations of MB. Furthermore, ROS generation assessed in confocal microscopy and spectrophotometry was reduced when the EAT and PVAT samples were acutely incubated with MB, as proof of the antioxidant effect of the redox dye in human adipose tissue. Last but not least, in the presence of serotonin, the MAO-A substrate, ROS production was further increased in both samples, an effect partially reversed by MB.

Besides inflammation, oxidative stress is also increased in the EAT but the sources of ROS are far from being fully elucidated. Salgado-Somoza et al. reported in their pioneering study that EAT produces a higher level of ROS than subcutaneous adipose tissue in patients with CHD. They found, among others, mRNA differences for catalase, glutathione S-transferase P, and protein disulfide isomerase [25]. Since EAT expands from epicardium into the myocardium, following the adventitia of the coronary arteries without any separation from myocardium, it provides harmful signaling via paracrine or vasocrine secretion and the oxidative stress in EAT may induce an increased oxidative stress in both coronary or myocardial tissue [26]. One year later, in EAT harvested from patients with severe stable CHD, Sacks et al. showed an increase mRNAs for 7 molecules involved in oxidative stress and/or oxygen species regulation along with 17 inflammatory adipokines or proteins involved in inflammation. One of the largest increases were reported for NADPH components gp91phox and p47 phox [27].

We have previously reported an increased oxidative stress in visceral adipose tissue (VAT) harvested from obese patients subjected to elective abdominal surgery and that MAO-A was the major isoform overexpressed. The fact that this finding was a particularity of obesity was further proven by the fact that ex vivo inhibition of MAO-A with clorgyline significantly reduced oxidative stress in VAT samples isolated from obese patients and had no effect in those harvested from the non-obese group [28].

The finding that MAO-A is also the predominant isoform in human EAT and PVAT is in line with the pioneering study published by Pizzinat et al. [29] in the late 90 s. Indeed, these authors reported that both MAO-A and MAO-B were expressed in human abdominal adipose tissue with MAO-A representing 70–80% of the total enzyme activity; also, the concomitant expression of noradrenaline transporter in human white adipocytes supports their role in the clearance of peripheral catecholamines. Our results confirm the fact that MAO-A is the predominant isoform in the diseased EAT (and PVAT) and its expression was mitigated by MB. We acknowledge as another limitation of the study the lack of use of MAO inhibitors in this study, in addition to MB, to (indirectly) assess the contribution of the latter to MAO inhibition.

PVAT is the fat depot surrounding most blood vessels, which in health presents anti-inflammatory and anti-contractile properties. At variance, in cardiometabolic pathologies associated with low-grade inflammation, PVAT-derived adipocytes generate various ROS, including superoxide anion and hydrogen peroxide that might signal to the vascular wall, underlying vascular injury. Classic sources of ROS in vascular beds include NADPH oxidase, uncoupled eNOS and dysfunctional ETS at the inner mitochondrial membrane [30, 31]. Moreover, superoxide is able to generate peroxynitrite (ONOO-) in the presence of NO and H_2_O_2_ can be converted into the highly reactive hydroxyl radical (–•OH) with further oxidation of lipids and DNA, thus leading to cell damage [30]. Interestingly, in healthy mouse mesenteric resistance arteries, it has been reported that PVAT acts as a reservoir for norepinephrine, preventing it from reaching the vessel and causing contraction [32].

We reported here that MAO-A is an important source of H_2_O_2_ in PVAT. We have also demonstrated that acute incubation of mesenteric arteries samples harvested form patients undergoing elective abdominal surgery with IL-6 increased MAO-A gene expression, as evidence of the fact that inflammation also potentiate the oxidative stress in the vascular wall [33]. Whether this observation can be recapitulated at the level of EAT and PVAT remains to be determined.

The first study reporting that MB is a potent reversible inhibitor of MAO-A was published almost two decades ago by Ramsay et al. These authors reported that MB, at concentrations reported to occur after intravenous administration, completely inhibited MAO-A (and partially MAO-B), due to its action as an oxidizing substrate and a one-electron reductant [34]. This MAO inhibitor effect, also common for other MB analogues [35], has been reported to mediate, at least partially, its antidepressant effects. Of note, the central inhibition of MAO-A by MB has also been linked to serotonin toxicity which may arise only when MB was used in combination with serotonergic drugs [36].

Methylene blue (MB) is known as a mild redox agent, which has been used as an electron carrier to prevent free radicals production and enhance cellular metabolic activity because it will not excessively accumulate in mitochondria and will not compromise the oxidation state of the physiological redox centers [37]. MB can reroute electrons in the ETS directly from NADH to cytochrome c, increasing the activity of complex IV activity and promoting ATP generation, while mitigating oxidative stress and delaying cellular aging by reversing neuroinflammation [38, 39].

The group of Adam–Vizi performed an elegant study aimed at elucidating the favorable energetic effects of MB in isolated guinea pig brain mitochondria treated with inhibitors of complex I or complex III of ETS. When the flow of electrons was compromised, MB transferred electrons to cytochrome c, increased the rate of ATP production, restored mitochondrial membrane potential, and improved the rate of calcium uptake. In rat heart mitochondria isolated from healthy and 2 months (streptozotocin-induced) diabetic rats, we have also demonstrated that addition of MB (0.1 μmol·L^−1^) elicited an increase in oxygen consumption of mitochondria energized with complex I and II substrates. In our hands, MB elicited a significant increase in H_2_O_2_ release in the presence of complex I substrates (glutamate and malate), but had an opposite effect in mitochondria energized with complex II substrate (succinate) [13].

More recently, the group of Mariana Rosca showed, in isolated diabetic cardiac mitochondria harvested from mice treated orally with MB that the redox agent facilitated NADH oxidation, increased NAD^+^, the activity of deacetylase sirtuin 3, and reduces protein lysine acetylation. Thus, by providing an alternative route for mitochondrial electron transport, MB alleviated the metabolic inflexibility in the diabetic heart [40].

MB has been extensively studied for its neuroprotective effects in animal models and patients with neurodegenerative diseases, in particular with Alzheimer disease, by targeting several molecular pathways that ultimately protect the brain mitochondria (comprehensively reviewed in ref. [41]). As an antidepressant, MB has been reported to act via various mechanisms. Accordingly, it restores mitochondrial function by acting as an alternative electron acceptor/donor, enhancing mitochondrial respiration, improving energy production and inhibiting the formation of superoxide. Also, MB has been also acknowledged as a non-selective inhibitor of NOS and modulator of the nitric oxide cyclic guanosine monophosphate (NO-cGMP) cascade, which enhances its antidepressant response, since dysfunction of the NO-cGMP cascade is involved in the neurobiology of mood, anxiety, and psychosis [42].

Recently, Pluta et al. [43] reported the successful reversal of vasoplegic shock by MB and ascribed the effect to the selective inhibition of iNOS (the inducible form of nitric oxide synthase), which prevented vasodilation in response to the pro-inflammatory cytokines.

We showed here, for the first time, that MAO is expressed in the human EAT and cardiac PVAT and MB, in submicromolar concentrations, can mitigate the oxidative stress in these two types of adipose tissue involved in the pathophysiology of cardio-metabolic diseases. Whether MB might reduce oxidative stress when administered as adjunctive pharmacological therapy during revascularization procedures, and improve the outcome of CHD patients with/without DM worth further investigation.

Pharmacological targeting of the epicardial and perivascular adipose tissues signaling pathways will remain a potential disease modifying approach in cardiometabolic syndromes. Whether MB will find a place in this scenario remains to be confirmed by future clinical studies.

## Conclusion

MAO-A expression and ROS generation are increased in the epicardial and perivascular adipose tissues harvested from overweight and obese patients with heart failure with preserved and mildly reduced ejection fraction. Methylene blue is able to reduce both MAO expression and the oxidative stress. Further studies are required to elucidate the signal transduction of these observations.

## Data Availability

No datasets were generated or analysed during the current study.
